# Secularism as a human right: learning from the European Court of Human Rights

**DOI:** 10.3389/fsoc.2024.1423747

**Published:** 2024-06-13

**Authors:** Haldun Gülalp

**Affiliations:** Independent Researcher, Istanbul, Türkiye

**Keywords:** secularism, separation of state and religion, freedom of religion, human rights, European Court of Human Rights

## Abstract

Secularism is conventionally (and somewhat misleadingly) defined as the separation of state and religion. This article offers an alternative and more refined concept of secularism as a normative political principle of social peace within the context of diversity. The argument that secularism, so understood, lies at the core of a notion of human rights, contra the critique it has been receiving in recent decades as being hostile to freedoms, is assessed conceptually and supported by an analysis of how it is (indirectly) articulated in the jurisprudence of the European Court of Human Rights.

## Introduction

The title of this article may sound surprising to some readers, considering that many detractors of secularism associate it with authoritarianism and condemn it as opposed to freedoms and democracy. Here I argue, however, that a state’s adoption of the *normative principle of secularism* (to be clearly defined below) and structuring its institutions accordingly, is a necessary condition for the protection of human rights. To understand the significance of this point, we must first dispose of the confusing definitions that are in general circulation. Currently, the most familiar formula for *secularism* (not to be confused with the sociological theory of *secularization*, see Gülalp, 2022) is the separation of the governance of the worldly affairs from the other-worldly; simply, the separation of state and religion. To cite a commonplace source, according to *Wikipedia* for example (accessed on 11 April 2024), “Secularism is most commonly thought of as the separation of religion from civil affairs and the state.” But, I submit, this is not the sole (or *essential*) characteristic of secularism. Though ultimately misleading, this definition nonetheless originates from the experiences of those states that first established secularism in the modern period.

## Historical background

The First Amendment (1791) to the United States Constitution of 1788 states: “Congress shall make no law respecting an establishment of religion, or prohibiting the free exercise thereof; or abridging the freedom of speech, or of the press; or the right of the people peaceably to assemble, and to petition the Government for a redress of grievances.” The first clause, which protects freedom of religion, is known as the “wall of separation” between state and religion. According to it, the state cannot establish or adopt a religious organization, discriminate between religious organizations, or prohibit belief and worship. In the early modern era, the philosophical foundation of the separation of church and state was laid by Protestantism, which opposed the temporal power of the Catholic Church and the bogus claim of its clergy to mediate the road to heaven, by affirming that religious belief is a matter of personal bond between God and the individual believer. This notion was subsequently acknowledged by Enlightenment thinkers (particularly Locke, 1689) as a condition of individual freedom of belief and tolerance between people of different faiths. The (liberal) principle of separating religious power from political power, that is, in essence purifying public-political issues from religious issues and stripping them of the possibility of domination by religious authorities, was adopted to attain this condition. The USA was founded under the leadership of Protestants and supporters of the European Enlightenment. The phrase itself, that is the “wall of separation” between church and state, was popularized by Thomas Jefferson, one of the “founding fathers,” in explication of the provision in the first article of the *Bill of Rights* (i.e., the First Amendment), and enjoyed wide circulation as an expression of US secularism.

We also see the concept of separation between state and religion in the title of France’s 1905 law, often considered a standard reference for secularism. While the first article of the French Constitution declares, among other things, that the country is secular (*laique*), respectful of all faiths, and that all citizens are equal before the law, regardless of origin, race or religion, the term “secular” or “secularism” (*laicité*) does not appear anywhere in the 1905 law. The subject of the law is quite literally the separation of religion and state, and its title includes the phrase “Law on the Separation of Churches and State” (*Loi concernant la séparation des Eglises et de l’Etat*). The reason why the law acquired this name and its related content is that from the 1789 revolution until 1905 the state experienced various forms of relationship with the churches, including seizing them and undertaking to pay the salaries of the clergy (Bauberot, 1998). The 1905 law authorized the solution reached at the end of more than a century of struggle for finding a final settlement. For France, the “separation of state and religion” meant that, following the turbulent period after the revolution, the republic had finally removed itself from under the influence and authority of the Catholic Church and established clear boundaries. The second article of the same law, in line with its counterpart in the US constitution, provides that “The Republic does not recognize, support, or subsidize any religion. Accordingly … all expenses relating to the exercise of worship will be removed from the budgets of the State, departments, and municipalities.” A further clause in the same article exempts some state institutions that provide public services, such as hospitals, schools, prisons, nursing homes, etc., so that “expenses relating to chaplaincy services … [in them] may be entered in said budgets,” as a condition of the freedom of belief.

On the other hand, we see elsewhere in Europe and in other parts of the world that the relations between the state and religious organizations are regulated in various ways without harming the essence of the principle of secularism. For example, several European countries, such as the United Kingdom, Denmark, and Norway, have an official state church; but this has mostly a symbolic value, while the states are essentially secular. In Germany, which does not have an official religion, the state may collect taxes from citizens on behalf of the church they belong to.

## Secularism and freedom of religion

How this “religion vs. state” problematic, arising from the specific experience of some Western countries, has shaped the more abstract and general definition of secularism is manifested in the objections raised to the concept in recent decades. Both fundamentalists and anti-secular postmodernists complain about the modern state’s intervention in religious political movements and organizations, arguing that secularism is an oppressive regime that restricts freedom of religion (e.g., Asad, 2003). The objection is often expressed as follows: If secularism is the separation of state and religion, then any intervention of the state will mean a violation of its own principle; yet modern states routinely intervene in religious affairs and do so in the name of secularism. For the state to *intervene* ostensibly to *separate* religion and state is proof that secularism is not only anti-democratic but also self-contradictory. It is thus an unsound objective and ought to be abandoned.

In fact, this objection itself is unreasonable, for it is impossible completely to separate religion and state. Every state, whether religious or secular, must have a policy regarding religion(s). Through legislation and bureaucratic procedure, it must establish a pattern of relationship with religious communities, whether to suppress, control, or support their organizations and activities. State policy may aim to confine or release religious entities, or may discriminate between them and treat them differently. Therefore, in and of itself, the state’s pursuit of a policy of relating to religious formations cannot be a criterion for defining (or condemning) secularism. Moreover, the democratic state has the authority and indeed duty to oversee all kinds of organizations and movements, religious or otherwise, and to regulate them in case they violate democratic rules. In a democracy, no organization can consider itself exempt from the rules. There is therefore no contradiction or logical inconsistency in the state’s pursuit of a policy on religion(s) to realize the normative principle of “secularism”.

There is a second, and more fundamental, aspect in the standard definitions of secularism that underlines the state’s impartiality in realizing freedom of religion and conscience. *Wikipedia*, again, offers the following second definition: “Secularism can be also defined as treating every religion equally and providing equal facility.” In other words, secularism involves both freedom of religion for everyone and equal treatment toward everyone regardless of religion, that is, no discrimination on account of religious belief or lack thereof. The institutional separation between church and state may even be considered only a “means” for these latter “ends” (Maclure and Taylor, 2011). This core aspect of secularism does not imply that the state should completely abandon relations with religious communities and formations; on the contrary, it imposes a positive duty on the state. An exemplary content of this duty may be found in the French law of 1905. The first article of the law guarantees freedom of religion and conscience, but also adds that some restrictions may be imposed on the use of this freedom in the name of public interest: “The Republic protects freedom of conscience. It guarantees the free exercise of religion subject only to the restrictions set out below in the interest of public order.” These restrictions are not open-ended. Although many articles of the law (as implied in its title) deal with arrangements about the state’s regulation of church properties and budgets, the last few articles clearly indicate what kind of restrictions may apply on the freedom promised in the first article. For example, actions that may call for intervention include political propaganda in places of worship, the spreading of teachings that promote hatred or violence, and the attempt to bring people into or out of any religious community by force or threat. The aim here is to protect the freedom of religion and conscience specified in the first article of the law equally among citizens. Likewise, the US Constitution’s Bill of Rights stipulates that the state cannot take sides between religions and cannot prevent worship.

## International human rights documents

This issue is expressed in similar ways in international documents on human rights. Article 10 of the *Declaration of the Rights of Man and of the Citizen*, associated with the French Revolution of 1789, contains the following statement: “No one shall be molested on account of his opinions, including his religious views, provided their manifestation does not disturb the public order established by law.” A very similar rule is included in Article 18 of the *Universal Declaration of Human Rights*, adopted by the United Nations in 1948: “Everyone has the right to freedom of thought, conscience and religion; this right includes freedom to change his religion or belief, and freedom, either alone or in community with others and in public or private, to manifest his religion or belief in teaching, practice, worship and observance.” This article has been transferred verbatim to the first clause of Article 9 of the *European Convention on Human Rights*, adopted by the Council of Europe in 1950; and the second clause of the same article defines the limits to the freedom in question: “Freedom to manifest one’s religion or beliefs shall be subject only to such limitations as are prescribed by law and are necessary in a democratic society in the interests of public safety, for the protection of public order, health or morals, or for the protection of the rights and freedoms of others”.

Attention may be drawn to some important points in these documents. First, freedom of religion and conscience, like any other freedom, is not indefinite within the bounds of democratic society. According to the most fundamental norm of the philosophy of freedom, if freedoms are to be equally valid for everyone, a person’s freedom cannot be at the expense of the freedom of another. Second, these documents place conscience and religion on a par with *thought*, which is clearly a this-worldly activity (Bielefeldt, 2013). This means that one is perfectly entitled to hold any belief they wish, but that belief does not occupy a higher plane than any thought that someone else may have. We usually tend to judge thoughts as right or wrong in our own opinion and may criticize or debate them; but when it comes to faith, we tend to stay away because it is considered sacred. Unlike thought, articles of faith may indeed be sacred; but they are so only to the believer. They, or belief in their sanctity, concern the believer alone and may not be imposed on others. By the same token, while one must feel no obligation toward a belief one does not share, one is still obligated to avoid humiliating, insulting, or mistreating a believer because of their belief. There is, in other words, the obligation to respect the other person’s freedom of belief.

Finally, freedom of religion means that individuals have the right to practice religious rituals, worship, and instruction, either alone or with others, in public or in private; but it does not mean that religious communities, organizations or their leaders have the right to access opportunities to dominate the public and political sphere. This is because, by their very nature and structure, religious organizations involve a hierarchy of authority based on absolute truths rather than discussions and negotiations that form the basis of democracy. Opening such a door to the public and political sphere will generate pressure on members of society who belong to different faiths or to no faith at all. The secular state, however, is obligated to ensure that every member of society feels equally free, safe, and entitled. It is due to this obligation that the state may need to intervene in religious organizations, communities, or their leaders.

## Secularism defined

These considerations lead us to a definition of secularism that is more comprehensive and refined than the concept of “separation of religion and state”: Secularism is a normative political principle that aims to guarantee citizens the right to freedom of conscience and religion, as spelled out in international human rights documents, and as such entails the existence of a political space separate from and independent of religions for the purpose of negotiating common issues and areas of concern, so that the social and political needs of all religious and irreligious members of society may be met. Secularism is an institutional framework that is not about endorsing or rejecting religion, but about protecting the freedom of thought, belief, and conscience of every citizen, regardless of whether they have religious beliefs or not. The aim of this protection is not to allow religion to occupy the political space, but to allow individuals to hold a belief and to fulfill its requirements individually or collectively, provided they do not violate the rights and freedoms of others.

A democratic state is equally liable toward all its citizens, whether religious or not, while religious communities and organizations have no such obligation. Therefore, it is the responsibility of the democratic state to ensure that all individuals and institutions, including religious ones, operate within the limits determined by the requirements of human rights and social peace. There is no good reason for religious organizations to have any immunity or differential status. A religious organization may not be granted a special position in worldly affairs, which properly fall within the scope of duty of the democratic state, beyond those activities that are related to faith and conscience, such as organizing worships and teaching tenets to believers. Hence, secularism is an institutional arrangement that separates politics from religion *in order to* guarantee the individual’s right to freedom of thought, conscience and belief. As a normative principle, it aims to ensure peace and tranquility, and is thus a foundation of free and equal citizenship in a society with diversity of beliefs.

## The European Court of Human Rights

The European Court of Human Rights (ECtHR), responsible for confirming whether the member states of the Council of Europe comply with the provisions of the European Convention on Human Rights has the authority, like any other court, to elaborate and clarify the provisions of the convention through its case-law. The ECtHR strives to preserve and develop its own jurisprudence by citing past decisions and judgments in every new case that it hears. Still, it is not always consistent, significantly because of its structural features. As some examples below will reveal, although the ECtHR has the natural tendency to develop a common language and culture through practice as a court, the occasional propensity of judges from different countries to act as representatives of their governments or their experience of having been socialized in different political cultures may be observed in many cases. In addition, given that it is ultimately an inter-state institution, situations may arise where it is difficult to interfere in the affairs of a powerful state, thus creating handicaps for individual applicants. Finally, the ECtHR, like all institutions, has the motivation to protect itself. For example, the growing burden created by the endless accumulation of applications may lead the Court to take some shortcuts or to turn a blind eye to the shortcuts that may be taken by the states that are the objects of litigation so that potential applications are prevented at the point of origin (Kurban, 2020). Regardless, the case-law developed over numerous cases alleging violation of Article 9 heard by the ECtHR gives us important insights as to how best to delineate the concept of secularism, confirming the definition we just proposed.

In its first-ever judgment that Article 9 of the Convention has been violated by a member state, the ECtHR clarifies the content of the article in question with the following observation: “As enshrined in Article 9, freedom of thought, conscience and religion is one of the foundations of a ‘democratic society’ within the meaning of the Convention. It is, in its religious dimension, one of the most vital elements that go to make up the identity of believers and their conception of life, but it is also a precious asset for atheists, agnostics, skeptics and the unconcerned. The pluralism indissociable from a democratic society, which has been dearly won over the centuries, depends on it.” (*Kokkinakis v. Greece,* No: 14307/88, 25 May 1993, Paragraph 31).

The cases judged by the ECtHR on Article 9 since then and the academic evaluations of these judgments are too numerous to properly address here. The ECtHR, which repeats this first comprehensive determination on freedom of religion and conscience in almost every judgment and decision on the same subject, also frequently repeats another finding that parallels the assessment we made above on the conceptual definition of secularism. In a formulation based on earlier findings but laid out clearly for the first time in *Leyla Şahin v. Turkey* (No: 44774/98, Fourth Section Chamber, 29 June, 2004; Grand Chamber, 10 November 2005), the ECtHR maintains that it is neutral, even indifferent, on the matter of how relations are structured between state and religion: “Where questions concerning the relationship between State and religions are at stake, on which opinion in a democratic society may reasonably differ widely, the role of the national decision-making body must be given special importance … Rules in this sphere will consequently vary from one country to another according to national traditions and the requirements imposed by the need to protect the rights and freedoms of others and to maintain public order … Accordingly, the choice of the extent and form such regulations should take must inevitably be left up to a point to the State concerned, as it will depend on the specific domestic context.” (Section Chamber, 2004, Paragraph 101; Grand Chamber, 2005, Paragraph 109).

As regards the problematic aspects of the functioning of the ECtHR, briefly mentioned above, some examples come to mind. In *Kokkinakis v. Greece*, the Greek member of the Court was not the only judge who voted against the majority ruling that the Greek government had violated the freedom of religion of Kokkinakis, a Jehovah’s Witness, who was prosecuted and sentenced for the offense of “proselytism.” Nevertheless, the Greek judge’s dissenting opinion sounded as if he were the lawyer of the Greek government rather than a member of the Court. While defending the ban on religious propaganda (which necessarily applies to beliefs other than Orthodox Christianity as the established religion of Greece), which was at the source of the case, the judge defined the act of proselytism as the “*rape* of the beliefs of others” (*italics added*). He concludes his opinion with the following remarks: “it is regrettable that the above judgment should allow proselytizing activities on condition only that they should not be ‘improper’. Can a convention on human rights really authorize such an intrusion on people’s beliefs, even where it is not a forceful one?” But that is precisely the gist of the matter. Manifesting a belief or thought or trying to persuade others in one direction or another is an integral part of freedom, so long as it does not involve any force or coercive threat.

In those Article 9 cases where the litigants bring the question of “secularism” into the debate and wish to oppose or defend it as a principle in their application, the ECtHR acts timidly and retreats behind the “margin of appreciation” of the states, that is, it gives “special importance” to “the role of the national decision-making body,” as quoted above. Such retreat then causes some inconsistencies between different judgments. A striking example in this regard is the contrast between the cases of *Leyla Şahin v. Turkey*, cited above, and *Lautsi v. Italy* (No: 30814/06, Second Section Chamber, 3 November 2009; Grand Chamber, 18 March 2011), which we examine next.

## ECtHR on secularism

In the case of *Leyla Şahin v. Turkey*, the petition filed for the applicant, who claimed that she was deprived of university education because she wears a headscarf, was concerned more with questioning and condemning Turkey’s constitutional principle of “secularism” than with the protection of an individual’s human rights (Gülalp, 2019, pp.151–154; see also Çakır, 2000, on how during the 1990s Islamists manipulated the headscarf issue for political advantage at the expense of the women who suffered the consequences). Both the Chamber of the Fourth Section and the Grand Chamber, which took the case upon the applicant’s objection, concluded that there was no violation, citing the following reasons: the constitution of the Republic of Turkey embodies the principle of secularism, the relevant regulations in universities and other public institutions are based on various national legislations, and all of these have been made public and were known to the applicant.

In *Lautsi v. Italy*, the applicant claimed that as the separation of church and state and freedom of belief are clearly protected in the Italian constitution, the presence of crucifixes on the walls of classrooms is a violation of the constitution and of the Article 9 rights of her two children who are not raised as Christian, as well as their educational rights protected by Article 2 of Protocol No.1 to the Convention (protecting the right of parents to have their children educated in conformity with their religious and philosophical convictions). In its judgment in favor of the applicant in 2009, a Chamber of the Second Section concluded that the pupils’ rights were indeed violated as alleged. The case was then brought to the Grand Chamber upon the objection of the Italian government, and with the support of several religious organizations and member states that carry a religious identity (such as Greece, Bulgaria, Armenia), which were given leave to “intervene” in the case. Ruling in 2011, the Grand Chamber reversed the lower chamber’s judgment. It reasoned that “secularism” is not a value to be protected and the figure on classroom walls represents a “historical tradition” rather than a “religious symbol.” Therefore, the Court reasoned, the issue of violating Article 9 does not even need to be evaluated, and the issue of the right to education is irrelevant, as what matters is the content of the curriculum and not the “passive” figures on the walls. Clearly, the striking contrast between the outcomes of these two cases has much to do with the application of the “margin of appreciation” doctrine, which means giving priority to the preferences of the state, sometimes interpreted too broadly by the Court (Gülalp, 2013; see also Lewis, 2007, and Berry, 2017).

The ECtHR, which may be indifferent on the question of separation between religion and state and somewhat inconsistent on the question of secularism in the abstract, is rather sensitive when it comes to beliefs and religions (especially minority ones) that states do not recognize or treat in a discriminatory manner (*cf.* Scolnicov, 2010). The ECtHR did not rule in favor of the applicant in the Leyla Şahin case, which brought Turkey’s constitutional principle of “secularism” into the debate; but it has persistently ruled for violations in response to a variety of cases against Turkey in applications made by *Alevi* individuals and organizations. These applications, which litigate against the consequences of the non-recognition of Alevism by the state, constitute a wide and interesting spectrum, as we see next.

## ECtHR on freedom of religion

In the case of *Hasan and Eylem Zengin v. Turkey* (No: 1448/04, 9 October 2007), regarding the content of compulsory religion courses in secondary education as problematic for Alevis, the ECtHR concluded that Article 2 of Protocol No. 1 to the Convention (right to education) was violated. It also ruled that the violation of the right to education continued in the case of *Mansur Yalçın and Others v. Turkey* (No: 21163/11, 16 September 2014), which alleged that there was no serious improvement in the content of the textbooks. In the case of *Sinan Işık v. Turkey* (No: 21924/05, 2 February 2010), filed by an Alevi citizen who did not want to have *Islam* written in the “religion” section of his identity card, the Court ruled that Article 9 was violated and stated that the violation was not due to what was written in the religion section, but essentially due to the presence of a religion box on identity cards. In the case of *CEM Foundation v. Turkey* (No: 32093/10, 2 December 2014), the complaint was that while mosques, churches, and synagogues as places of worship were exempt from electricity bills, *cemevis* (where Alevis perform collective worship) were deprived of this right because the state did not recognize them as such. In this case, the ECtHR found that Article 14 of the Convention, which prohibits discrimination, was violated in conjunction with Article 9. Significantly, the Court did not make a judgment as to whether the electricity bills of religious organizations or places of worship should be covered by the state (i.e., it did not focus on the issue of separation between religion and state), but emphasized that it was wrong for the state to discriminate between religious beliefs without relying on an objective and reasonable justification. Finally, in the case of *İzzetin Doğan and Others v. Turkey* (No: 62649/10, 26 April 2016), the complaint was that the Presidency of Religious Affairs of the government provided public services only to citizens of the Sunni sect of Islam and disregarded those who adopted the Alevi faith. This case was directly heard by the Grand Chamber, which ruled that both Article 9 and Article 14 (taken in conjunction with Article 9) had been violated.

Two interesting points deserve attention in the detailed reasoning of the judgment in this latest and very comprehensive case. First, the Court notes that they do not interfere with the issue of how religion-state relations are structured, but focus on whether the state discriminates between communities in providing services: “The Court stresses that its task in the present case is not to ascertain whether the requests made by the applicants should or should not have been granted, particularly since they related to a large number of spheres. Furthermore, it is not the Court’s place to impose on a respondent State a particular form of cooperation with the various religious communities. As already stated …, there is no doubt that the States enjoy a margin of appreciation in choosing the forms of cooperation with the various religious communities. However, whatever form is chosen, the State has a duty to put in place objective and non-discriminatory criteria so that religious communities which so wish are given a fair opportunity to apply for a status which confers specific advantages on religious denominations…” (Paragraph 183).

Secondly, and perhaps more interestingly, in this case heard by 17 judges, several judges voted against the majority decision that there had been a violation of Article 9, and they did so for similar reasons. Among them, the Dutch judge (Johannes Silvis) expressed his objection most succinctly: “There is no obligation under the Convention for the State to seek an active supporting role in matters of religion. For that reason I respectfully disagree with the majority that there has been a violation of Article 9 taken alone.” In other words, according to this judge, and the others who expressed their objections in their own words, the state’s failure to support the religious affairs of the Alevis cannot be considered a violation of the freedom of religion and conscience, protected by Article 9; the problem is merely discrimination, i.e., the violation of Article 14 (taken in conjunction with Article 9). Considered collectively, the joint opinion of the dissenting judges is that the correct way to eliminate the violation in question is *not* to provide the services enjoyed by the Sunnis to the Alevis as well, but to eliminate such a duty of providing service altogether.

## Secularism as freedom of religion

Based on the above, we may also reassess the extent to which the constitutional classification of Turkey as “secular,” characterized (and condemned) by Islamist and some multiculturalist circles as being too “radical,” is a valid description (Gülalp, 2022). We have already seen that the concept of separation between religion and state, as one of the possible (and widely circulating) definitions of secularism, is quite meaningless. Even if it were meaningful, this definition would still not apply to Turkey, considering the existence of the Presidency of Religious Affairs as a government bureaucracy, especially given the extraordinary size of its budget and personnel. Some writers who criticize secularism in general, and Turkey’s “secularism” in particular, prefer to define the concept not as the separation of religion and state, but rather as the state’s *domination* over religion, such that the state uses its power to try and determine the place and the boundaries of religion in society, to distinguish between “good” and “bad” (or right and wrong) religions, and to accordingly shape the religious life and worldview of its citizens (Azak, 2010). In this view, the problem with secularism in Turkey is not in the establishment of links between state and religion but in the presumed “oppression” of religion by the state. If the state pursued a policy of *supporting* religion by providing space in political life for religious communities, organizations, and their leaders, then the degree of secularism would be diminished, its sharp edges softened, and it would be turned into a “moderate” secularism, which enhanced freedom of religion and conscience (Modood, 2019). But the fallacy of this conceptualization may readily be seen by considering, for example, the religious policy of the Ottoman Empire, which cannot be described as secular by any (modern) criteria and cannot be said to have protected the freedom of religion and conscience of its “citizens” (or *subjects*, to use an historically more accurate term). The Ottoman state defined and classified religion into “right” and “wrong,” discriminated even between those classified as “right,” and regulated religious life and relations between religious communities through the bureaucratic office of the *Şeyhülislam* (directly controlled by the Sultan), which was the institutional predecessor of the Presidency of Religious Affairs of the republican period.

I suggested above that a more comprehensive and meaningful definition of secularism could be offered, and that it would contain two critical elements: that the state protects the freedom of thought, conscience, and religion of its citizens and that it does so equally, without discrimination. Clearly, Turkey has problems in both regards (freedom and equality) and hence a meaningful or proper kind of secularism is non-existent, despite the appearance of this term in various articles of the constitution. According to Article 2, the Republic is “secular.” Article 10 stipulates that “Everyone is equal before the law without distinction as to language, race, color, sex, political opinion, philosophical belief, religion and sect, or any such grounds,” and according to Article 24, “Everyone has the freedom of conscience, religious belief and conviction.” References to secularism also appear in too many other articles to be mentioned here. Despite all this, however, the actual absence of secularism (properly defined) is not simply a current problem that originates from the policies of an Islamist government, as one might be tempted to believe, but an inherent feature of the political culture, exacerbated indeed in the current period. A comparison of a case judged by the Turkish Constitutional Court (TCC) with an ECtHR case, which originated from the Netherlands on a nearly identical issue, will reveal how the dominant political culture in Turkey treats freedom of religion and equality, irrespective of what appears in the text of the constitution.

Before a detailed examination of these two cases, we may briefly describe how secularism is affirmed in the Netherlands. The first article of the constitution states that “All persons in the Netherlands shall be treated equally in equal circumstances. Discrimination on the grounds of religion, belief, political opinion, race or sex or on any other grounds whatsoever shall not be permitted.” Article 6 stipulates that “Everyone shall have the right to profess freely his religion or belief, either individually or in community with others, without prejudice to his responsibility under the law.” The second clause of the same article states that “Rules concerning the exercise of this right other than in buildings and enclosed places may be laid down by Act of Parliament for the protection of health, in the interest of traffic and to combat or prevent disorders.” Finally, according to the third clause of Article 23, “Education provided by public authorities shall be regulated by Act of Parliament, paying due respect to everyone’s religion or belief.”

## Turkish Constitutional Court on the call to prayer

In 2008, the applicant D.Ö. (prudently remaining anonymous) complained that his sleep was disturbed by the call to prayer recited loudly in the mosques near his house in the early morning hours and that he felt forced to worship the Sunni sect to which he did not belong. He applied to the local Administrative Court in Izmir, where he lived, requesting an end to the procedure and non-pecuniary damages. The Court noted that the administration had issued circulars regarding the sound level and was properly implementing them, as documents in the file showed that the sound system was inspected. Moreover, the Court added, the call to prayer does not mean forcing non-believers to worship, so there is no discrimination. The plaintiff then took the matter to the Council of State, which confirmed in 2013 the decision of the first court, and finally to the Turkish Constitutional Court in 2014, which also reached the same conclusion in 2016 (TCC, *D.Ö.*, No: 2014/3977, 30/6/2016).

The applicant claimed before the TCC that the volume of the sound interrupted his sleep and disturbed him, and that he was forced to worship a sect to which he did not belong. Moreover, he added, most of the people living in the neighborhood did not go to mosque, and yet the state carried on with this procedure. The state is also obligated to ensure that people live in a healthy and peaceful environment, and yet it neglected this duty. Insisting on this practice violated the principles of protecting private life, freedom of religion and conscience, secularism, and equality. The plaintiff claimed that the sum of violations concerned the following articles of the Constitution: Article 2 (secularism); Article 5 (peace and happiness of society); Article 10 (equality before the law); Article 12 (everyone has fundamental rights and freedoms); Article 13 (restricting these rights and freedoms cannot be contrary to democracy and secularism); Article 24 (freedom of religion and conscience); Article 56 (protection of health and the environment); Article 136 (Presidency of Religious Affairs operates in accordance with the principle of secularism). In short, the plaintiff’s complaint was based on three issues: Secularism (Articles 2, 13, 24, 136), equality (Articles 10 and 12), and health and the environment (Article 56).

The Ministry of Justice, asked by the TCC for an opinion, responded as follows: “In our country, where the majority is Muslim,” the call to prayer is a practice shared by all sects of Islam and has no discriminatory aspect. It “has been internalized by many segments of society and become a part of their culture.” Finally, “it is necessary to compare the discomfort brought on individuals by the events complained about and described as noise with the benefit aimed to be achieved in the continuation of the religious practice accepted by the majority of society, and establish a fair balance [between them].”

Several elements in this response deserve attention: The generalization that “all sects of Islam” share the call to prayer excludes the Alevi faith, which has its own set of worship practices and yet is considered by many of its followers to be a belief system within Islam. Similarly, the statement that the call to prayer has been “internalized by many segments of society” leaves out non-Muslims and non-believers. Moreover, this statement is an assertion that could not have any legal status, being based on a superficial impression (what exactly does “many segments” mean?) and ignores the problem of the high decibel of the sound, which is the main subject of the complaint. The importance of this assertion is that it lies at the basis of the thesis that brings the Ministry’s assessment to a conclusion. The Ministry asks what is more important: the discomfort felt by a random individual about the “events” that he describes as “noise,” or the practice that is already accepted (more accurately, *assumed* to be accepted) by the majority of society?

After conveying the views of the parties to the case, TCC moves on to its own assessment and begins with a surprising determination. Although the plaintiff (rightly or wrongly) claimed that a total of eight articles of the constitution were violated, the Court ignores these claims, by citing from a previous ruling that “The Constitutional Court is not bound by the applicant’s legal characterization of the events and makes its own legal description of the events and the facts,” and chooses to assess the case within the scope of Article 17 (“Everyone has the right to life and the right to protect and improve his/her corporeal and spiritual existence”), which was not even among those mentioned by the plaintiff (Paragraph 23). This choice is explicated as follows (from Paragraph 24 on): The applicant’s sole concern is being woken up from sleep in the early hours, which has nothing to do with the freedom of religion and conscience. Besides, sounding the call to prayer does not prevent anyone from performing their alternative form of worship. The applicant cannot explain how he has been discriminated against. Therefore, there is no point in considering this application within the scope of articles that concern secularism and equality. The only question that needs to be examined is whether there is a threat to the corporeal and spiritual existence of the person, constitutionally protected by Article 17.

The questionable character of this procedure aside, the Court nonetheless proceeds to make a detailed assessment of Article 56 (from Paragraph 31 on), which was initially left out of consideration. The Court notes that this article finds meaning within the context of Article 8 of the European Convention (“Right to respect for private and family life”), and concludes that there is no violation in this regard, because urban life necessarily entails the kinds of disturbances that the applicant complains of, which are relatively “insignificant” (Paragraph 37). The Court additionally notes that the general deterioration of the environment, noise, and similar problems cannot be considered as violations, because the “Convention does not guarantee the right to live in a clean and quiet environment” and therefore “it is not possible to examine within the scope of Article 8 of the Convention such environmental rights that have no bearing on legal interests as the right to have a nice view or live in a nice surrounding” (Paragraph 38).

Following these considerations, which do not directly concern the issue at hand, the Court pronounces its final decision: First, to evaluate the environmental impact of the sound that the applicant complains about, its “intensity, duration, and physical and psychological effects” must be assessed (Paragraph 41). However, the applicant “has not included in his application any concrete data such as the measurement of the volume of the sound, and the distance of the audio device to the residence and the angle [at which it stands]” (Paragraph 45). Second, “the individual’s right to protect his material and spiritual existence must be balanced with the interest of society in [the continuation of this practice] … which is a requirement of the belief of the majority” (Paragraph 45). Third, the call to prayer is an ancient “*religious ritual*” and a cultural value (Paragraph 50). Therefore, “democratic tolerance and pluralism make it inevitable to allow some practices in line with the belief of the majority of society and … create an obligation to tolerate [them]” (Paragraph 51). There may indeed be a violation if this obligation reaches unbearable proportions, but no violation has been found by the courts where the applicant sought his rights in this regard (Paragraphs 52–53). Therefore, the applicant’s assertion of violation is “unacceptable” (Paragraph 55).

The TCC rightly notes that the applicant’s petition does not contain any measurement or any other indirect evidence of the volume of the sound. Yet, this important deficiency notwithstanding, two points in the final judgment must be underlined. First, the Court defines the compliance of the rights-seeking individual with the majority as a requirement of pluralism, and places the burden of tolerance on the individual. However, pluralism and submission to the majority are opposite principles. The concept of human rights and the relevant conventions assign the duty of “democratic tolerance” to the majority and the state, not to the individual. Second, if the TCC, which cites many ECtHR cases in its long and detailed reasoning, were to refer to a parallel case heard by the ECtHR four years previously, it may have had to reach a different conclusion. Indeed, the ECtHR’s treatment of the matter, summarized below, constitutes a striking contrast to the TCC’s oxymoronic juxtaposition of pluralism with religious majoritarianism and its attempt to legitimize the latter under the pretext of democratic tolerance.

## The European Court of Human Rights on the church bell

H.C.W. Schilder was the priest of a Catholic church in Tilburg, Netherlands. He would ring the church bell for 3 min every morning at 7:15 a.m. to summon the congregation to the 7:30 a.m. service. In early 2007, people living in the neighborhood warned him that they were disturbed by the loud sound of the bell. The priest then reduced the ringing of the bell to 1 min, but did not lower its volume. The residents had also complained about the situation to the municipality. The local authorities came to the site to measure the volume of the sound; and finding that it was indeed too loud, they issued a warning that if the volume would not be reduced to a tolerable level, a fine of up to 5,000 Euros would be imposed for each violation, up to a maximum of 50,000 Euros. The parish board of the church refused to act on this order and instead filed a case with the Regional Court, complaining of the violation of their freedom of religion.

The Regional Court reached the following judgment within the same year: Ringing the church bell is within the scope of freedom of religion and attempting to restrict it constitutes an interference with this freedom. On the other hand, the municipality has the authority and responsibility to regulate the duration and volume of any sound that may disturb the residents. However, the existing legal provision that grants the municipality this authority does not include the sound of a church bell. Therefore, the church’s objection to the imposition of a fine is justified.

This judgment was not actually a negative outcome for the neighborhood and the municipality, it only pointed to the gap in the ordinances. Indeed, the local government did not appeal this judgment but instead amended the relevant provision to bring church bells and other forms of call to prayer into its scope of regulation. This amendment, which was made in 2008 and came into force in the following year (2009), mandated that between the hours of 23:00 and 7:30 the sound of church bells could not exceed the standard laid down by the Environmental Management Decree by more than 10 decibels. There would be no limitation for other hours.

With this new provision measurements were taken and warnings were issued to the church again, in response to which the church applied to the Regional Court again. This time, however, by a judgment issued in 2010, the Court rejected the church’s appeal. The Court ruled that the municipality had the authority to demand the lowering of the sound of the church bell between the specified hours, but that the church had failed to demonstrate that this could not be done. The church’s subsequent appeal to the Council of State was also rejected in 2011. Finally, the parish priest, representing the church, took the case to the ECtHR in 2012, claiming that his Article 9 rights were violated (*H.C.W. Schilder v. The Netherlands*, No: 2158/12, 16 October 2012).

In the ECtHR’s account, Priest Schilder argued before the Council of State that the Environmental Management Decree defined the night hours as being between 23:00 and 7:00, but the municipality arbitrarily set the time limit at 7:30. He also claimed that it would be necessary to make a special effort to lower the sound of the bell, perhaps without success. If successful, only a minority of the congregation would be able to hear the bell and many more would miss the service. Finally, he argued that the amended article did “not serve the municipal interest but only the special interests of complaining neighboring residents.” (Note the striking resemblance between the reasoning of the priest and that of the TCC, covered above.) The Council of State judged against this application on the following grounds: The municipality has the authority to regulate sound levels, including the sound of church bells. Ringing the church bells is a manifestation of belief, which is protected, but “this right does not imply the freedom to ring [them] … without any limits as regards duration and volume.” The municipality also has both the right to set 7:30 as the time limit and an interest in this limitation, which “lies concretely in the protection of the inhabitants of the municipality against disturbance of their nights’ rest.” Besides, “this provision does not make it impossible to ring the bells before 7.30 a.m. and no restrictions apply for the sound level of church bell ringing between 7.30 a.m. and 11 p.m.” Therefore, the Regional Court was correct in its findings (Paragraph 13).

The ECtHR reached its own decision on the application without dwelling much on it. The Court first considered whether the issue at hand concerned Schilder personally, so that he could be considered a “victim” within the meaning of the Convention, and ruled that the application being inadmissible at any rate for other reasons, this question was insignificant (Paragraph 16). The Court further ruled that, while Article 9 protects both belief and its manifestation, it “does not always guarantee the right to behave in the public sphere in a way which is dictated by such a belief. The term ‘practice’ in Article 9 does not cover any act which is motivated or influenced by a religion or belief” (Paragraph 18). The Court also noted, “In democratic societies, in which several religions and beliefs coexist within one and the same population, it may be necessary to place restrictions on the freedom to manifest one’s religion or belief in order to reconcile the interests of the various groups and to ensure that everyone’s beliefs are respected” (Paragraph 21). Finally, based on the observation that “the applicant was not subjected to a blanket ban on ringing the bells of his parish church but only to a restriction to the effect that during the hours of night … the ringing of the church bell should not exceed a defined volume … [and no] restriction … for the hours between 7.30 a.m. and 11 p.m.” (Paragraph 23), “the application must be rejected for being manifestly ill-founded” (Paragraph 24).

## Conceptual perspectives

We have seen that the ECtHR may become disoriented and produce inconsistent judgments when “secularism” is questioned and debated as an *ideology*, and simply ignores to treat state-religion relations as a matter of human rights, choosing to leave it to the national authorities of member states; but it remains on more solid footing when the issue at hand is an individual’s freedom of belief (*cf.* Evans and Thomas, 2006, for a similar observation). This is also how we argued “secularism” ought to be defined as a normative principle and that this definition ought to replace the more conventional one referring to the separation of state and religion. Ironically, what is ignored by the ECtHR is precisely the beginning point of much academic literature on secularism, particularly those that are critical of it. Writers who tend to malign secularism for its purported neglect of freedoms propose a range of (alas, unsatisfactory) concepts as to the “proper” relationship between state and religion.

In one such conceptual perspective, the question is raised if the state is “assertive” or “passive” in its dealings with religious affairs (Kuru, 2009). In this perspective, both Turkey and France are cases of “assertive” secularism, where the state tends “to exclude religion from the public sphere and confine it to the private domain,” while in the USA the state plays a “passive” role “by allowing the public visibility of religion” (Kuru, 2009, p.11). This means that the *quality* of secularism is measured by the “public visibility” of religion, i.e., the more the better. But while the public visibility of religion may benefit certain religious actors, no theoretical or empirical correlation may be established between such visibility and democracy or human rights more generally.

In another critique, the concept of state “neutrality” vis-à-vis religion is questioned. The argument is that if the modern nation-state shapes religion and religiosity via its sovereign power, it cannot be described as neutral; hence, the secular state’s claim to religious neutrality is an internal contradiction (Asad, 2006). But this argument misses the point that the state can never really be neutral vis-à-vis the religious affairs of its citizens. As already mentioned, the state must surely have some kind of policy, whether detrimental to or supportive of religious freedoms. The real issue for secularism is whether the state is neutral *between* religions in society. In other words, if for any reason there is need for intervention in religious affairs, it should not discriminate in favor of one (or several) over the others.

But then this principle of neutrality between all beliefs or religions is precisely violated by the notion of “multiculturalism,” yet another alternative advanced by some critics (Modood, 2019), because it implies the espousal of some specific religious communities, necessarily at the expense of others, as “multi” does not denote an unlimited number. Secularist neutrality between religions would necessarily allow for religious diversity in society, rendering void the need for (and hence the concept of) multiculturalism. If the state did not concern itself with the variety of beliefs in society, real diversity would be achieved not only among different religious communities but also among individual believers within each community. The liberal-democratic state should clearly opt for the protection of the freedom of the individual citizen, for a religious community typically has its own system of hierarchical authority and limits to internal diversity.

Finally, the concept of “twin tolerations” between church and state has been offered as an alternative (Stepan, 2000). But that too is problematic, because, again, as already pointed out, a democratic state is answerable to *all* citizens, whether religious or not, or whether adherent of this religion or that, whereas religious communities and their organizations are not. It is therefore the democratic state’s responsibility to ensure that all persons and institutions, including the religious ones, operate within limits set by the requirements of human rights and social peace.

The problem with all these critiques of secularism is that they confuse (or conflate) freedom *for* religion with freedom *of* religion. The former is a mode of granting certain privileges to religious actors, while the latter is a mode of granting freedoms and equality to (believing and non-believing) individuals.

An alternative conception, at first sight supportive of secularism and resembling the argument advanced in this article, duly emphasizes that “secularism is not against religiosity, but fiercely opposes institutionalized religious domination” (Bhargava, 2013, p.81). But, while the line of reasoning in this conception begins with the hardly disputable observation that we ought to “jettison the standard church-state models and focus instead on secularism as a response to religious diversity” (p.80), it ends up with the notion of “principled distance” between the state and religion(s), which not only retains the church-state framework but further diversifies and complicates the links between the two, contingent on the priorities of the state and each religious community (p.86). The notion of “principled distance” promotes an even deeper involvement of the state in religious affairs, selectively designed for each religion (or the concrete religious community). A selective state policy of “supporting” or “inhibiting” the practices of different religious communities may ensue, depending on the details of each case and justified by the principle that different needs may necessitate differential treatment. Secularism in this sense is no longer a rule-based normative principle; it is “contextual,” bordering on what may be described as strategic manipulation by the state. Moreover, this notion unwittingly ignores the declared objective of preventing intrareligious (as well as interreligious) inequality and domination, because it still classifies people into religious communities and identifies individuals in relation to them.

## Conclusion

The ECtHR may not have a clear and consistent concept of “secularism,” but it does have a clear and (relatively) consistent concept of freedom of religion. The approach of the Court relies on a basic principle that offers a way to cut through the myriad and confusing conceptualizations for a normative mode of state regulation of religion. It appears that from the ECtHR’s perspective, whatever the institutional structure may be, the crux of the matter is the observance of the right granted in Article 9 of the European Convention on Human Rights, which protects the “freedom of thought, conscience and religion.” Thus, while the court may reject an explicit position on state-religion relations, its case-law reveals an implicit position on what is essential in a coherent and meaningful concept of secularism.

The problem, I have argued, originates from the confusion about how to correctly define and conceptualize “secularism.” There is, however, a definition that would satisfy both “secularists,” who insist on the creation of separate spaces for religion and politics, and those who insist on freedom of religion, but only insofar as that freedom is granted to individual citizens and not to (bureaucratic or communal) religious actors, provided they respect the rights of others just as their own rights are to be respected. Basically, secularism is a formula for social peace in a society composed of a variety of believers and non-believers, whereby individuals have the freedom of thought and conscience as specified for protection by international covenants; and the only way to guarantee this is through the constitution of a political space that is separate from and independent of religions. The secular state is obligated to secure both freedom of belief and the limits that can rightfully be imposed on this freedom. As such, far from being oppressive or restrictive of freedoms, secularism stands at the very center of the concept of rights-bearing citizenship.

## Author contributions

HG: Writing – original draft, Writing – review & editing.

## References

[ref1] Asad T. (2003). Formations of the secular. Stanford, CA: Stanford University Press.

[ref2] Asad T. (2006). “Trying to understand French secularism,” in Political theologies: Public religions in a post-secular world, eds Vries H. de Sullivan L. E. New York, NY: Fordham University Press, 494–526.

[ref3] Azak U. (2010). Islam and secularism in Turkey. London: I.B. Tauris.

[ref4] Bauberot J. (1998). “Two thresholds of laicization” in Secularism and its critics. ed. Bhargava R. (Oxford: Oxford University Press), 94–136.

[ref5] Berry S. E. (2017). Religious freedom and the European court of human rights’ two margins of appreciation. Rel. Hum. Rights 12, 198–209. doi: 10.1163/18710328-12231145

[ref6] Bhargava R. (2013). Reimagining secularism: respect, domination and principled distance. Econ. Polit. Wkly. 48, 79–92.26663948

[ref7] Bielefeldt H. (2013). Misperceptions of freedom of religion or belief. Hum. Rights Q. 35, 33–68. doi: 10.1353/hrq.2013.0009

[ref8] Çakır R. (2000). Direniş ve İtaat: İki İktidar Arasında İslamcı Kadın. Istanbul: Metis.

[ref9] Evans C. Thomas C. A. (2006). Church-state relations in the European court of human rights. BYU Law Rev. 3, 699–725.

[ref10] Gülalp H. (2013). “Religion on my mind: secularism, Christianity and European identity” in Religion, identity and politics: Germany and Turkey in interaction. eds. Gülalp H. Seufert G. (London: Routledge), 164–179.

[ref11] Gülalp H. (2019). Religion, law and politics: the ‘trickle down’ effects of ECtHR judgments on Turkey’s headscarf battles. Rel. Hum. Rights 14, 135–168. doi: 10.1163/18710328-13021148

[ref12] Gülalp H. (2022). Secularism as a project of free and equal citizenship: reflections on the Turkish case. Front. Sociol. 7:902734. doi: 10.3389/fsoc.2022.902734, PMID: 35782710 PMC9240275

[ref13] Kurban D. (2020). Limits of supranational justice: The European court of human rights and Turkey’s Kurdish conflict. Cambridge: Cambridge University Press.

[ref14] Kuru A. (2009). Secularism and state policies toward religion: The United States, France, and Turkey. Cambridge: Cambridge University Press.

[ref15] Lewis T. (2007). What not to Wear: religious rights, the European court, and the margin of appreciation. Int. Comp. Law Quarter. 56, 395–414. doi: 10.1093/iclq/lei169

[ref16] Locke J. (1689), A letter concerning toleration, (various editions).

[ref17] Maclure J. Taylor C. (2011). Secularism and freedom of conscience. Cambridge, MA: Harvard University Press.

[ref18] Modood T. (2019). Essays on secularism and multiculturalism. London: ECPR Press.

[ref19] Scolnicov A. (2010). “Does Constitutionalisation Lead to secularisation? The case law of the European court of human rights and its effect on European secularisation” in Religion and the political imagination. eds. Katznelson I. Jones G. S. (Cambridge: Cambridge University Press), 295–313.

[ref21] Stepan A. (2000). Religion, democracy and the ‘twin tolerations’. J. Democr. 11, 37–57. doi: 10.1353/jod.2000.0088

